# B-cell-depletion reverses dysbiosis of the microbiome in multiple sclerosis patients

**DOI:** 10.1038/s41598-022-07336-8

**Published:** 2022-03-08

**Authors:** Alba Troci, Olga Zimmermann, Daniela Esser, Paula Krampitz, Sandra May, Andre Franke, Daniela Berg, Frank Leypoldt, Klarissa Hanja Stürner, Corinna Bang

**Affiliations:** 1 Institute of Clinical Molecular Biology, Kiel University, Kiel, Germany; 2 Department of Neurology, Kiel University, Arnold-Heller-Strasse 3, 24105 Kiel, Germany; 3 Institute of Clinical Chemistry UKSH Kiel/Lübeck, Kiel University, Kiel, Germany

**Keywords:** Neuroimmunology, Applied microbiology, Computational biology and bioinformatics, Multiple sclerosis

## Abstract

To elucidate cross-sectional patterns and longitudinal changes of oral and stool microbiota in multiple sclerosis (MS) patients and the effect of B-cell depletion. We conducted an observational, longitudinal clinical cohort study analysing four timepoints over 12 months in 36 MS patients, of whom 22 initiated B-cell depleting therapy with ocrelizumab and a healthy control group. For microbiota analysis of the oral cavity and the gut, provided stool and oral swab samples underwent 16S rDNA sequencing and subsequent bioinformatic analyses. Oral microbiota-patterns exhibited a reduced alpha-diversity and unique differential microbiota changes compared to stool such as increased levels of Proteobacteria and decreased abundance of Actinobacteria. Following B-cell depletion, we observed increased alpha-diversity in the gut and the oral cavity as well as a long-term sustained reduction of pro-inflammatory Gram-negative bacteria (e.g., *Escherichia/Shigella*). MS patients have altered stool and oral microbiota diversity patterns compared to healthy controls, which are most pronounced in patients with higher disease activity and disability. Therapeutic B-cell depletion is associated with persisting regression of these changes. Whether these microbial changes are unspecific side-effects of B-cell depletion or indirectly modulate MS disease activity and progression is currently unknown and necessitates further investigations.

## Introduction

With an estimated number of more than 2 million people worldwide, Multiple Sclerosis (MS) is the most prevalent chronic inflammatory disease of the central nervous system (CNS) affecting primarily young females^1,2^. In recent years, the importance of the gut microbiome as an indicator and mediator of inflammation has been discovered in several inflammatory diseases^3^. Consequently, several studies have elucidated the gut microbiota in MS patients (reviewed in^3^) and identified a number of alterations of microbial composition in diseased subjects (reviewed in^4^). Herein, plausible immunological links between these differences and MS pathogenesis were found^3^. Thus, there is increasing evidence that an altered microbiome composition might influence the progression of MS—however data on longitudinal persistence of microbial changes, differences between the oral and aboral microbiota and the effect of immunotherapy on microbial composition is still lacking. This lack of data on oral microbiota is remarkable since impaired oral health of MS patients has already been described 30 years ago^5^ and there is increasing evidence that bacteria of the oral cavity can directly influence neuro-immune activity and inflammation^6,7^.

Due to the high efficacy in controlling inflammation in MS by the monoclonal B-cell-depleting antibody ocrelizumab (Ocrevus^®^) for relapsing–remitting MS, we initiated a longitudinal, observational study recruiting patients with planned B-cell depletion therapy and age- and gender-matched patients who decided against immunotherapy.

## Results

**Figure 1:**
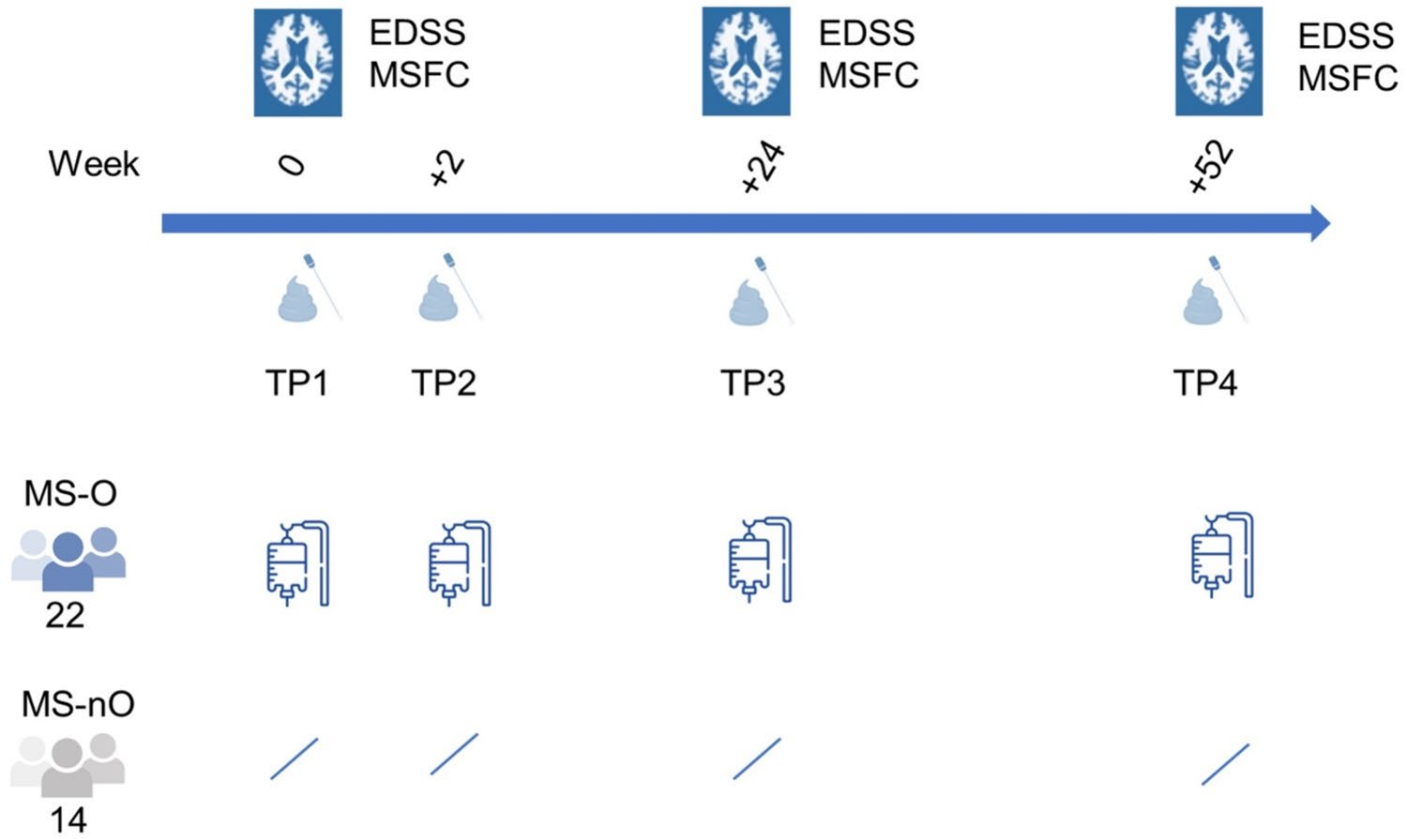
Study design. 37 MS patients, of whom 22 initiated B-cell depletion therapy with ocrelizumab and 14 age- and gender-matched untreated MS patients were recruited for this observational study. Four timepoints (days 0 and 14, weeks 24 and 52) over 12 months were analysed including microbiota analysis of provided stool and oral swab samples as well as clinical outcome and MRI results.

We recruited 22 patients who had decided to start treatment with ocrelizumab (MS-O) and 14 age- and gender- matched, currently untreated MS patients (MS-nO). Demographic and clinical data are presented in Table 1, the study design is shown in Fig. 1. Despite similar disease duration, untreated patients had significantly better scores for EDSS and MSFC at baseline (Table 1). None of the patients treated with B-cell depletion (MS-O) had new T2-lesions at week 52, increased EDSS score or worsened MSFC-scores (Supplemental Fig. S1). Only one of the non-treated MS patients (MS-nO) had new T2-lesions at week 52 and only one patient from the MS-O and MS-nO group, respectively changed their diet habits during the study.

**Table 1:** Baseline characteristics in the untreated and ocrelizumab-treated MS patient cohort.

	Untreated MS patients (n = 14)	Ocrelizumab-treated MS patients (n = 22)
Age (years; median with range)	46.5 (29–65)	45 (21–56)
Women	12 (85.7%)	18 (81.8%)
Time since diagnosis (years; median with range)	6.5 (1–37)	7 (0.5–27)
RRMS/SPMS (SPMS in %)	4/10 (28.6%)	6/16 (27.3%)
EDSS at baseline (median with range)	1.0 (0.0 – 3.5)	3.75 (1.0 – 6.5)
**MSFC at baseline (median with range)**		
T25 (s)	4.56 (2.95 to 6.9)	7.05 (3.8 to 26.2)
TTW (s)	8 (4.6 to 17.5)	14.8 (6.1 to 45)
9HPT (s; dominant hand)	19.2 (17.4 to 21.6)	26.2 (17 to 42.8)
SDMT (standard deviation)	0 (− 1 to + 1.5)	− 0.5 (− 3 to + 2.5)
Number of patients without previous MS-treatments	12 (85.7%)	4 (18%)
Received disease-modifying therapies previously	2 (14.3%)	18 (81.8%)
		Natalizumab: 7 (31.8%) Dimethylfumarate: 10 (45.5%) Injectables: 11 (50%)
At least 2 previous therapies	0 (0%)	15 (68.18%)
At least 3 previous therapies	1 (7%)	11 (50%)
Time without disease-modifying therapy before start of B-cell depletion (months; median with range)	–	3.5 (1–15)
Reported periodontitis	4	7

Absolute numbers with percentages or range in brackets.

### Differences in oral and stool microbiota composition of healthy controls and MS patients

**Figure 2:**
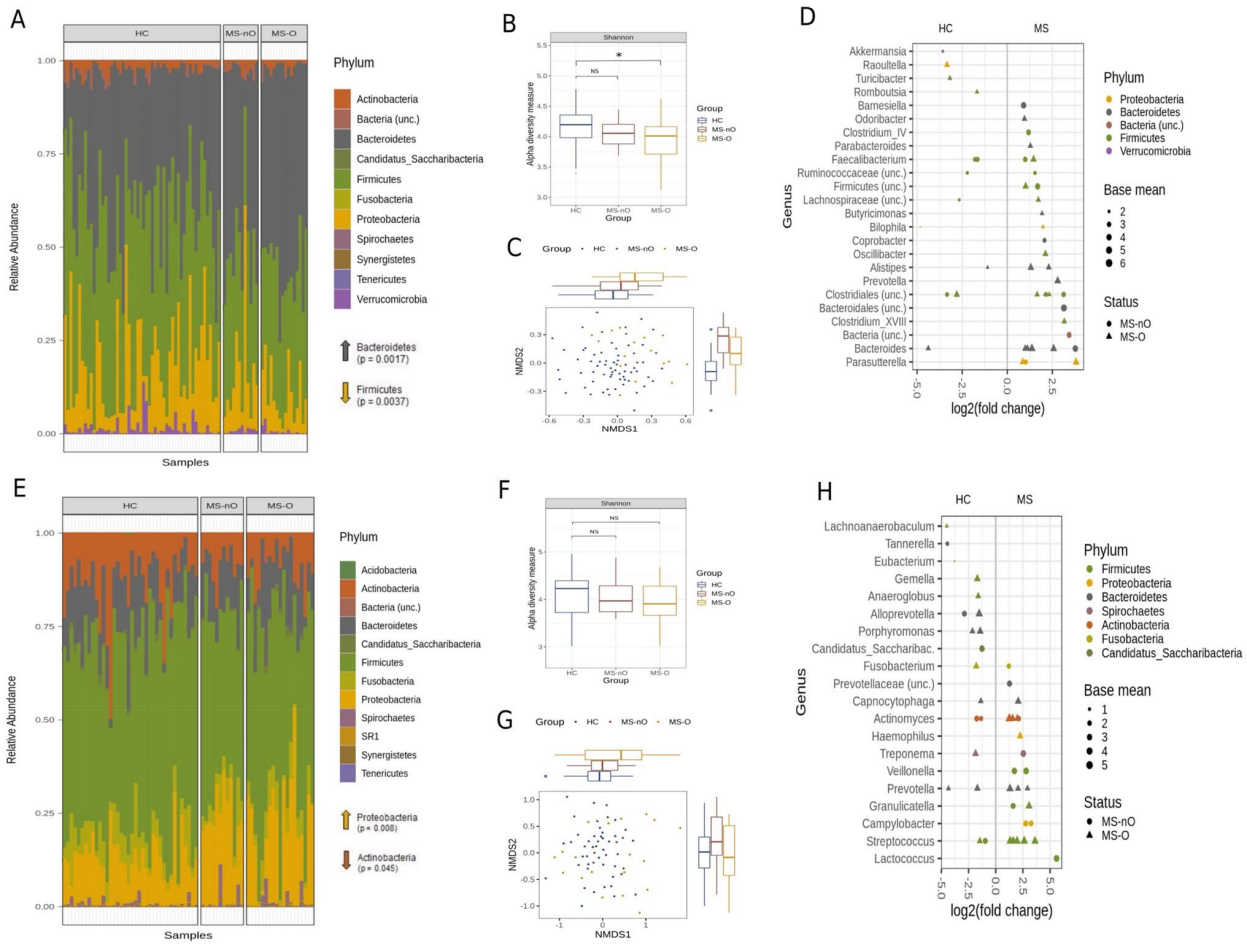
Gut and oral microbiota analysis of healthy controls and MS patient groups at baseline. Total abundance of bacterial phyla in samples from healthy controls and MS patients revealed shifts in phyla composition of stool samples (**A**) as well as in oral swabs (**E**). Alpha diversity measurements for MS patients (at baseline) versus healthy controls showed a more pronounced decrease for the MS-O group in stool (**B**) and swab samples (**F**). Beta-diversity was analysed based on Bray–Curtis distance matrices of microbial communities in all samples and visualized using non-metric multidimensional scaling (NMDS). Patients with MS (red dots) showed a shift indicating compositional differences to healthy controls, which were significant for both MS groups in both sample types (**C** stool; **G** swab samples). Significant abundant taxa between MS patients and healthy controls in stool (**D**) and swab samples (**H**) were calculated by using GLMM (at least 50 read pairs/sample, prevalence ≥ 5%). The right part of the plots shows taxa, which were more abundant in MS patients and in the left part those with higher abundance in healthy controls. Dot/triangle colours indicate phyla affiliation of shown genera and dots are specific for MS-nO whereas triangles represent MS-O group.

First, baseline stool microbiota of both MS patient groups included in this study were compared to microbiota profiles from healthy controls (HCs) (Supplemental Table S1). Total abundance of bacterial phyla identified in stool revealed significant higher abundance of Bacteroidetes in MS patients compared to HCs (*p* = 0.0017) as well as significant lower abundance for Firmicutes (*p* = 0.0037) (Fig. 2A). These differences were mainly driven by the clinically more severely affected MS-O patient group [Fig. 2A, Supplemental Table S2, Bacteroidetes: HC vs. MS-O (*p* = 0.0017), HC vs. MS-nO (*p* = 0.29), Firmicutes: HC vs. MS-O (*p* = 0.044), HC vs. MS-nO (*p* = 0.112)]. Next, we analyzed the diversity and evenness of microbial composition (alpha diversity) in MS patients versus HCs We observed a generally lower alpha diversity in stool of MS patients that was again found to be more pronounced in MS-O patients (Fig. 2B, pShannon HC vs. MS-O = 0.017, pShannon HC vs. MS-nO = 0.109). Regarding overall bacterial composition and diversity, the MS patient groups showed a general shift indicating compositional differences between MS patients and HCs (Fig. 2C) [pAdonis = 0.001 (pAnosim HC vs. MS-O = 0.036, pAdonis2 HC vs. MS-nO = 0.002)). Significant differentially abundant taxa between MS and HCs are shown in Fig. 2D (*p* < 0.05). As already observed at phyla level, in MS patients, and in particular in the MS-O group, a general higher abundance of several Bacteroidetes members could be observed (esp. the genera *Prevotella*, *Butyricimonas*, *Coprobacter*, *Parabacteroides* and *Alistipes*), while on the other hand members of the phylum Firmicutes were frequently less abundant (e.g., members of the Clostridiales as well *Romboutsia* and *Turicibacter*). Compared to HCs, both patient groups revealed a significant higher abundance of *Parasutterella* (Fig. 2D).

Secondly, the oral microbiota profile of MS patients was compared to that of HCs at baseline. Total abundance of bacterial phyla in the oral cavity is shown in Fig. 2E and revealed significant higher levels of Proteobacteria in MS patients (*p* = 0.008), whereas Actinobacteria were found to be decreased (*p* = 0.045). In contrast to the findings from stool, these results were mainly driven by the less affected MS-nO patient group [Fig. 2E, Supplemental Table S2, Proteobacteria: HC vs. MS-O (*p* = 0.122), HC vs. MS-nO (*p* = 0.002), Actinobacteria: HC vs. MS-O (*p* = 0.255), HC vs. MS-nO (*p* = 0.127)]. Alpha-diversity was slightly, but not significantly decreased in MS patients versus HCs (Fig. 2F). With respect to overall bacterial composition, significant differences between MS patients and HCs were observed (pAdonis = 0.002) (Fig. 2G), which could also be verified in each of the MS patient groups and their respective HCs (pAnosim HC vs. MS-O = 0.001, pAdonis HC vs. MS-nO = 0.02). Significant different abundant taxa between MS and HCs are shown in Fig. 2H (*p* < 0.05). MS patients generally had significant higher abundances of the genera *Campylobacter*, *Haemophilus* and *Neisseria*—all belonging to the Proteobacteria, though this was only partly evident when splitting up the MS patients in groups (Fig. 2H).

### Oral and stool microbial changes after B-cell depletion

**Figure 3:**
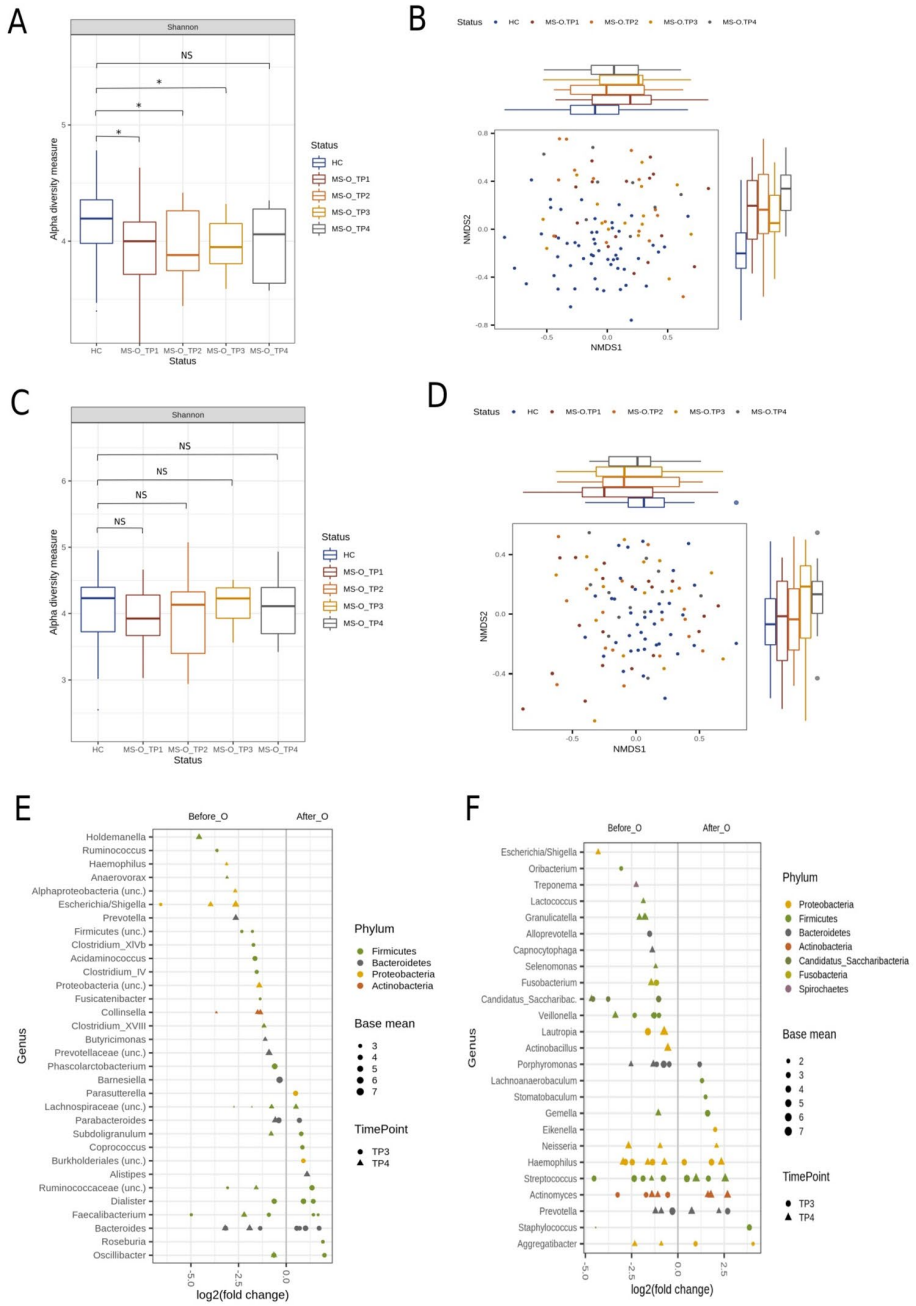
Microbial changes in the stool and oral cavity under treatment with ocrelizumab. Alpha diversity measurements (by Shannon) for MS-O patients showed a slight, but not significant, increase in stool (**A**) and swab samples (**C**). Compositional changes of stool (**B**) and oral (**D**) microbiota were visualized using NMDS. Differentially abundant taxa in MS patients before and after treatment with ocrelizumab in stool (**E**) and oral swab samples (**F**) were calculated by using a generalized linear mixed model (GLMM) after 6 and 12 months. In the right parts of the plots, taxa are more abundant in MS patients before treatment and in the left parts, taxa are more abundant after treatment. Dots represent changes after 6 months, whereas triangles correspond to changes after 12 months. Dot/Triangle colors indicate phyla affiliation of shown genera.

When comparing microbial changes in MS-O patients during treatment, results from fitted models displayed a general slight increase in alpha diversity between HCs and MS patients before (TP1) and during treatment (TP2, TP3, TP4) in stool (Fig. 3A) and swab samples (Fig. 3C), though none of these changes was found to be significant (Supplemental Table S3). However, species richness determined by Chao1 index was significantly increased in swab samples at TP4 (pChao1 = 0.025) (Supplemental Tables S3 and S4). Regarding beta diversity, a non-significant shift between MS patients at baseline compared to MS patients 6 months (TP3) and 12 months (TP4) after B-cell depletion in both stool and swab samples was observed (Fig. 3B, D, E; Supplemental Tables S3 and S4). Comparing the compositional differences in the oral cavity to HCs at TP1, the initially observed highly significant difference (pAnosim = 0.001) became less at TP4 (pAnosim = 0.023).

Next, we determined fine-scale abundance differences over time in treated patients (MS-O). We concentrated on changes persisting over an extended 6-months period (TP3-TP4, months 6–12) following B-cell depletion and found a significant decrease of members of the phylum Proteobacteria (particularly members of the genus *Escherichia/Shigella*) as well as varying genera from the Bacteroidetes (*p* < 0.05, e.g., *Bacteroides*, *Parabacteroides* and *Prevotella*; Fig. 3C) in stool samples. In contrast, numerous members of the Firmicutes were altered differentially (e.g., *Faecalibacterium, Dialister* and *uncl. Ruminococcaceae*), however were still less abundant compared to HCs. Within the oral cavity, several bacterial genera were consistently altered at 6- and 12-months following B-cell depletion (Fig. 3F). We found a significant decrease of members of the genera *Haemophilus* and *Lautropia* out of the Proteobacteria, *Porphyromonas* out of the Bacteroidetes, *Veillonella* and *Granulicatella* out of the Firmicutes as well as unclassified members from the phylum *Candidatus* Saccharibacteria. Different ASVs of the genera *Streptococcus* and *Actinomyces* were consistently increased following treatment.

## Discussion

Key findings of our study are (I) the specific microbial fingerprint observed in oral swabs in MS patients, (II) the stool microbial changes associated with disease severity at baseline and (III) the persisting long-term reversibility of these compositional differences following B-cell depletion.

So far, there is no common type of dysbiosis described for the stool microbiota of MS patients^4^. Though in general, a reduced alpha-diversity has been observed throughout most studies, inconsistent results regarding specific changes of microbial species have been described so far. As reviewed during the last years^4,8,9^ studies on gut microbial changes in MS are characterized by relatively low proband numbers from different geographical areas that are often highly diverse in their disease status as well as in their medication. In addition, distinct sequencing and analysis strategies were used that hamper overall comparison of results. In accordance with that conclusion, the results of our study do resemble some earlier findings of an altered bacterial composition in the gut, whereas others are striking: particularly, the overall observed decrease of Firmicutes with members of the *Clostridiales* was described in various MS patient cohorts earlier^10–12^. Many bacterial members of this group are involved in the production of short-chain fatty acids, particularly butyrate, and are thus implicated with anti-inflammatory characteristics crucially involved gut homeostasis (reviewed in^13^). The observed significant increase of several Bacteroidetes in the MS patient cohort has not been described so far^14^. In our patient collective especially members of the *Porphyromonadaceae* (e.g., *Parabacteroides*), of the *Rikenellaceae* (e.g., *Alistipes*) and unclassified members of Bacteroides have been found to be significantly enhanced in MS patients with more severe disease courses. The observed significant higher abundance of the genus *Parasutterella* has also been associated with dysbiosis and a lack of diversity in the microbial stool composition in inflammatory bowel disease^15^. Thus, in general, the stool microbiota of MS patients does resemble earlier findings of a more pro-inflammatory composition containing higher proportions of gram-negative bacteria possibly triggering inflammation via production of lipopolysaccharide that activates TLR4 signaling^14,16^.

Interestingly, after treatment of MS patients with ocrelizumab, the described pro-inflammatory dysbiosis changed. We detected an overall decrease of Bacteroidales in the treated patients, particularly with respect to the abundance of *Parabacteroides*, *Bacteroides* and *Prevotellaceae*. In addition, several members of the Proteobacteria (including genus *Escherichia/Shigella*) decreased massively. These observations are in line with previous studies of stool microbiota changes under anti-inflammatory treatment, e.g., in IBD^17^ but also in MS^11^ and other conditions^16^ and thus strongly underline the intensive crosstalk between microorganisms in the gut and inflammatory conditions of the host. In a recent study analyzing inter alia the influence of disease-modifying treatments including B-cell depletion in cross-sectionally recruited MS patients (n = 25 receiving B-cell depleting therapies)^18^, basically β-diversity did not differ between treated or untreated MS patients, while MS disease status had a relevant effect on the microbiota composition compared to healthy controls. Changes for *Ruminococcae* and *Lachnispirae* were identified in this cohort for B-cell-depleted MS patients, too, however, probably due to the analyses of prospectively and at harmonized time-points longitudinally collected samples the effect of B-cell depletion on microbiota composition in our cohort appears to be more distinct or better distinguishable.

Whereas the stool microbiota has gained much interest in chronical neuroimmune diseases during the last years, the oral microbiota has only rarely been investigated, particularly in MS patients. Given the fact that a link has already been made between Alzheimer’s disease as well as autism spectrum disorders and several specific microbes of the oral cavity and the well-known poor oral health of many MS patients, this is astonishing^5–7^. Thus, during this current study, we sampled and analyzed oral swabs from MS patients and found a significant higher abundance of members of the Proteobacteria, especially *Haemophilus*, *Neisseria* and *Campylobacter*. Whereas all of these species are known to be part of the oral microbiota^19^, the higher proportion of those gram-negative bacteria in MS patients is noteworthy. All of those genera have related species causing severe diseases in humans^20^. Particularly, for non-typeable *Haemophilus influenzae* a connection to disease etiology of MS has already been discussed due to their expressed cell-surface N-glycosylated adhesins, which are considered as native antigen potentially be involved in triggering pathogenic antibodies in MS^21^. In contrast to the data observed in the stool microbiota, changes in the oral cavity did not correlate clearly with severity of disease, potentially excluding a generalized effect of inflammatory systemic processes. However, additional data on the oral microbiota of MS patients in general are necessary to understand the overall role of *Haemophilus* and other members of the Proteobacteria in disease manifestation and progression.

After treatment of MS patients with ocrelizumab we noticed a bidirectional exchange of several specific members of different genera including *Prevotella*, *Actinomyces*, *Streptococcus* and *Haemophilus* that might resemble the high variation of those strains in the oral cavity in general. In addition, we observed a significant decrease of some members of the so-called red complex (*Treponema denticola*, *Porphyromonas gingivalis)*, which are associated with severe forms of periodontal disease^22^ and thus might be involved in the described overall poor oral health of MS patients. Interestingly and in agreement with findings from the stool microbiota a decrease of the genus *Escherichia/Shigella* particularly after 12 months could also be observed, again arguing for its potential role during inflammatory processes.

Though the patient numbers are rather small in this study, the strength of it is its longitudinal design, its appropriate definition of age- and gender matched disease cohorts with healthy controls as well as the inclusion of oral microbiota analyses. However, about 80% of the included MS-O patients had a history of previous treatment with disease-modifying therapy potentially influencing microbiota composition. On the other hand, the inclusion of patients with mild and severe disease course allowed a more focused view on the manifested microbial changes during disease progression.

## Conclusions

We conclude that not only stool, but also oral microbiota composition is altered in patients with MS and that these oral findings represent a more distinct “disease trait” than the gut microbiota composition in MS. Furthermore, treatment with B-cell depleting antibodies in MS patients does not only reduce overall disease activity, but also associates with specific changes of stool and oral microbiota composition. Whether these therapy-associated changes are specific to B-cell therapies, were non-relevant side-effects or indirectly contribute to its effect remains to be elucidated. Our results need to be reproduced in cohorts with higher patient numbers, other immunosuppressive drugs, non-MS inflammatory controls, and with further deep sequencing analyses before any conclusions can be drawn and possible interventional trials designed.

## Methods

### Ethics

The study was conducted in accordance with the Declaration of Helsinki, and ethical approval was granted by the ethics committee at Kiel University (AZ A103/14, D440/18 and D498/19). All volunteers provided written informed consent before entering the study.

### Cohort sampling

MS patients between 18 and 65 years with an MS diagnosis according to the revised McDonald criteria^23^ were recruited at the neuroimmunological outpatient unit of the UKSH Kiel between February 2018 and March 2020. MS patients were either untreated for at least 12 months before entering the observational study or had decided to start treatment with ocrelizumab independently of their study participation. Patients were matched for age and gender in the untreated versus ocrelizumab-treated MS patient group and initially answered a standardized extensive health and nutritional questionnaire (FFQ) and short follow-up questionnaires at each timepoint to document nutrition and medication. Patients who did not sent in at least samples from baseline, week 2 and week 24 were excluded from analysis. Clinical examination and magnetic resonance tomography (MRI) took place at baseline and months 6 and 12. For microbiota analysis of the oral cavity and the gut, all patients provided stool and swab samples twice before the first administration of ocrelizumab (day 7 and day 0, timepoint (TP)1) 2 weeks after the first infusion (day 14, TP2), as well as 6 (TP3) and 12 (TP4) months later. Clinical, i.e. Expanded Disability Status Scale Scores (EDSS) and Multiple Sclerosis Functional Composite (MSFC) as well as MRI data during follow-up were regularly obtained from clinical routine for all participating MS patients.

Age- and gender-matched healthy controls (HCs) from the same geographic area were chosen from another study collective with identical phenotypic and lifestyle data and sample assessment (Supplemental Table S1).

### Stool and swab sample processing and sequencing

DNA of stool samples was extracted using the QIAamp DNA fast stool mini kit automated on the QIAcube (Qiagen, Hilden, Germany). Material was transferred to 0.70 mm Garnet Bead tubes (Dianova, Hamburg, Germany) filled with 1.1 ml InhibitEx lysis buffer. For swab samples QIAamp UCP Pathogen mini kit automated on the QIAcube was used. The swab was therefore transferred to a Pathogen Lysis Tube S filled with 0.65 ml ATL buffer (incl. DX) and incubated for 10 min at 56 °C with continuous shaking at 600 rpm. Bead beating for both sample types was performed using a SpeedMill PLUS (Analytik Jena, Jena, Germany) for 45 s at 50 Hz with subsequent continuation of the manufacturer’s protocol. Extracted DNA was stored at − 20 °C prior to PCR amplification. Blank extraction controls were included during extraction of samples.

Amplicon sequencing of variable regions 1 and 2 of the 16S rRNA gene was done as been described in detail earlier^24^.

### Sequence data processing

Data processing was performed using the DADA2 version 1.10^25^ workflow for big datasets (https://benjjneb.github.io/dada2/bigdata.html) resulting in abundance tables of amplicon sequence variants (ASVs) according to a workflow adjusted for V1–V2 region, which can be found here: https://github.com/mruehlemann/ikmb_amplicon_processing/blob/master/dada2_16S_workflow_with_AR.R). Resulting ASVs underwent taxonomic annotation using the Bayesian classifier provided in DADA2 and using the Ribosomal Database Project (RDP) version 16 release. One sample with less than 10,000 sequences was not considered for further analysis.

### Statistical analyses of microbiota analyses

The R package “vegan” was used to investigate alpha diversity in this study. Alpha diversity of the samples was measured by Shannon diversity. The association between microbial diversity and compared groups (HCs vs. MS patients or between time points) was tested via paired and unpaired Wilcoxon rank-sum test for nonparametric data and *t* test. *T*-tests were performed after visual data inspection by histograms and when normal distribution of data was given as tested by the Shapiro–Wilk test for normality.

For analysis on beta-diversity, non-metric multidimensional scaling (NMDS) was performed with Bray–Curtis dissimilarity as implemented in the R package vegan v2.5-1. Bray–Curtis distance matrices based on the microbial communities in all samples were generated using the R package phyloseq v1.22.3. A permutational multivariate analysis of variance (PERMANOVA) was then performed on the distance matrix to assess the effects of status (healthy controls vs MS patients or MS patients’ different points in time) on variance between microbial communities. The PERMANOVA was performed based on the Adonis function of the R package vegan v2.5-1, with 999 permutations.

A generalized linear mixed model (GLMM) was calculated for each ASV, which was covered by at least 50 read pairs and showed a minimum prevalence 5% in all samples, to estimate the bacterial abundance in relation to the respective condition (healthy controls vs MS patients or MS patients’ different points in time). Models were corrected for subject intervariability and sequencing read depth, allowing for interactions. We used the package glmmTMB to estimate the abundance of each microbe under a zero-inflated Negative Binomial distribution. For each predictor, ASVs were excluded where the method did not converge or the Akaike Information Criterion (AIC) for model quality was not defined. Multiple testing correction was calculated using the Holme’s procedure. Significant abundant taxa were visualized using ggplot.

## Supplementary Information

Supplementary Information.

## Data Availability

The datasets generated and analyzed during the current study are available at the European Nucleotide Archive (ENA, Project accession number: PRJEB44538), please see https://www.ebi.ac.uk/ena/browser/search. Codes are available via this link: https://github.com/trocialba/Multiple_Sclerosis_Study.
