# Supplementary material for: B-cell-depletion reverses dysbiosis of the microbiome in multiple sclerosis patients
Source: Sci Rep. 2022 Mar 8;12:3728. doi: 10.1038/s41598-022-07336-8 (PMC8904534; doi:10.1038/s41598-022-07336-8)
Supplement: Supplementary file 1 — Supplementary Information. [file 41598_2022_7336_MOESM1_ESM.docx]

**Supplement**

## **Supplementary Table 1**

## Demographic and clinical characteristics of the study populations.

| Group | HC | | Ocrelizumab (O) | | No Ocrelizumab (nO) | |
| --- | --- | --- | --- | --- | --- | --- |
|  |  |  | MS-O | | MS-nO | |
|  | Stool | Swab | Stool | Swab | Stool | Swab |
| Total nr | 54 | 38 | 22 | 22 | 14 | 14 |
| Gender (female) | 32 | 27 | 18 | 18 | 12 | 12 |
| Age.mean (95% CI) | 47 (44-50) | 44 (40-48) | 41 (37-46) | 41 (36-45) | 46 (40-53) | 46 (40-53) |
| BMI.mean (95% CI) | 25 (24-26) | 24.9 (24-26) | 24.7 (22-27) | 24.6 (22-27) | 24.6 (22-27) | 24.6 (22-27) |
| Smoking (yes) | 8 (14%) | 8 (21%) | 3 | 3 | 0 | 0 |
| Medication (yes) | 11 (20%) | 8 (21%) | 13 | 13 | 8 | 8 |
| Medication type | 10 mg Atorvastin, Cetirizin, Thyreoprevent, Metoprolol 47,5mg, Ibuprofen 600mg, Desogestrel, GingoRing, L-Thyrox, Vitamin D 1000 IE, Amlodipin 5 mg | 10 mg Atorvastin, Cetirizin, Thyreoprevent, Metoprolol 47,5mg, Ibuprofen 600mg, Desogestrel, GingoRing, L-Thyrox, Vitamin D 1000 IE, Amlodipin 5 m | Ocrelizumab, Vitamin D 1000 IE, Metoprolol 50mg | Ocrelizumab, Vitamin D 1000 IE, Metoprolol 50mg | Vitamin D 1000 IE, Metoprolol 50mg, Ramipril 5mg | Vitamin D 1000 IE, Metoprolol 50mg, Ramipril 5mg |

**Table S1:**  Total nr = Total number of probands and samples for all groups that were included in the microbiota analysis. In addition, baseline characteristics such as age, gender, BMI, smoking status and actual medication type is given.

**Suppl. Table 2    Phylum abundances in stool samples**

| **Healthy Controls versus all Multiple Sclerosis patients** | | | |  |  |  |  |  |
| --- | --- | --- | --- | --- | --- | --- | --- | --- |
| Phylum | Abundance | Prevalence | log2FoldChange | baseMean | Chisq | df | P | padj |
| Bacteroidetes | 601825 | 82 | 0.32146 | 8.94813 | 13.43733 | 1 | 0.000247 | 0.001727 |
| Firmicutes | 552018 | 82 | -0.20754 | 8.782672 | 11.70036 | 1 | 0.000625 | 0.003749 |
| Proteobacteria | 231188 | 82 | -0.33915 | 7.84599 | 3.268814 | 1 | 0.070609 | 0.282435 |
| Verrucomicrobia | 17195 | 61 | -0.79979 | 5.418472 | 5.071473 | 1 | 0.024323 | 0.121614 |
| Actinobacteria | 27003 | 82 | -0.31135 | 5.707413 | 2.54886 | 1 | 0.110374 | 0.331122 |
| Bacteria..unc.. | 14389 | 55 | 0.197841 | 5.539759 | 0.233 | 1 | 0.629308 | 0.629308 |
| Synergistetes | 123 | 5 | 1.014527 | 2.706919 | 2.525821 | 1 | 0.111997 | 0.331122 |
|  |  |  |  |  |  |  |  |  |
| **Healthy Controls versus untreated Multiple Sclerosis patients** | | | |  |  |  |  |  |
| Phylum | Abundance | Prevalence | log2FoldChange | baseMean | Chisq | df | P | padj |
| Bacteroidetes | 455634 | 66 | 0.245636 | 8.919798 | 3.538068 | 1 | 0.059975 | 0.299876 |
| Firmicutes | 463634 | 66 | -0.20408 | 8.79398 | 5.789749 | 1 | 0.01612 | 0.112839 |
| Proteobacteria | 202603 | 66 | -0.11512 | 7.967583 | 0.199668 | 1 | 0.654989 | 0.654989 |
| Verrucomicrobia | 15779 | 53 | -0.86563 | 5.39078 | 3.861195 | 1 | 0.049415 | 0.296491 |
| Actinobacteria | 22757 | 66 | -0.34189 | 5.701727 | 1.677243 | 1 | 0.195291 | 0.615733 |
| Bacteria..unc.. | 12708 | 46 | 0.602004 | 5.766656 | 1.387735 | 1 | 0.238788 | 0.615733 |
| Synergistetes | 109 | 4 | 1.141722 | 2.741387 | 2.032834 | 1 | 0.153933 | 0.615733 |
|  |  |  |  |  |  |  |  |  |
| **Healthy Controls versus Ocrelizumab-treated Multiple Sclerosis patients** | | | | |  |  |  |  |
| Phylum | Abundance | Prevalence | log2FoldChange | baseMean | Chisq | df | P | padj |
| Bacteroidetes | 469158 | 70 | 0.374794 | 8.904211 | 13.40786 | 1 | 0.000251 | 0.001754 |
| Firmicutes | 451707 | 70 | -0.21013 | 8.710787 | 7.169521 | 1 | 0.007415 | 0.044492 |
| Proteobacteria | 180397 | 70 | -0.54788 | 7.671041 | 5.904733 | 1 | 0.0151 | 0.075501 |
| Verrucomicrobia | 15059 | 51 | -0.72603 | 5.384365 | 2.182508 | 1 | 0.139587 | 0.558347 |
| Actinobacteria | 22113 | 70 | -0.28894 | 5.648048 | 1.688988 | 1 | 0.193734 | 0.581203 |
| Bacteria..unc.. | 10130 | 48 | -0.28725 | 5.212128 | 0.296276 | 1 | 0.586226 | 0.682875 |
| Synergistetes | 33 | 3 | 0.767971 | 2.535419 | 0.905027 | 1 | 0.341437 | 0.682875 |

**Suppl. Table 3 Phylum abundances in swab samples**

| **Healthy Controls versus all Multiple Sclerosis patients** | | | |  |  |  |  |  |
| --- | --- | --- | --- | --- | --- | --- | --- | --- |
| Phylum | Abundance | Prevalence | log2FoldChange | baseMean | Chisq | df | P-value | P adjusted |
| Firmicutes | 847095 | 69 | -0.05937 | 9.412899 | 1.058905 | 1 | 0.303465 | 1 |
| Actinobacteria | 194326 | 69 | -0.42763 | 7.891146 | 8.066766 | 1 | 0.004508 | 0.045085 |
| Proteobacteria | 283450 | 69 | 0.568781 | 8.263606 | 11.45701 | 1 | 0.000712 | 0.007835 |
| Fusobacteria | 90253 | 69 | -0.29309 | 7.21206 | 3.069173 | 1 | 0.07979 | 0.529775 |
| Bacteroidetes | 207788 | 69 | -0.04263 | 8.032643 | 0.095058 | 1 | 0.757843 | 1 |
| Candidatus_ Saccharibacteria | 10069 | 66 | 0.043857 | 5.079795 | 0.028847 | 1 | 0.865133 | 1 |
| Spirochaetes | 11769 | 55 | 0.78734 | 5.354094 | 3.333504 | 1 | 0.067882 | 0.529775 |
| SR1 | 1261 | 30 | 0.514697 | 3.740412 | 1.078006 | 1 | 0.299144 | 1 |
| Tenericutes | 1262 | 21 | 1.145819 | 4.041022 | 4.145032 | 1 | 0.041757 | 0.375812 |
| Synergistetes | 692 | 17 | 0.082739 | 3.81724 | 0.020245 | 1 | 0.886856 | 1 |
| Bacteria..unc.. | 494 | 21 | 1.132217 | 3.053382 | 3.37427 | 1 | 0.066222 | 0.529775 |
|  |  |  |  |  |  |  |  |  |
| **Healthy Controls versus untreated Multiple Sclerosis patients** | | | | |  |  |  |  |
| Phylum | Abundance | Prevalence | log2FoldChange | baseMean | Chisq | df | P-value | P adjusted |
| Firmicutes | 657170 | 50 | -0.08172 | 9.459306 | 1.198346 | 1 | 0.273652 | 1 |
| Actinobacteria | 155794 | 50 | -0.47784 | 7.923624 | 6.014404 | 1 | 0.01419 | 0.127706 |
| Proteobacteria | 196682 | 50 | 0.636948 | 8.35527 | 14.05728 | 1 | 0.000177 | 0.001951 |
| Fusobacteria | 73285 | 50 | -0.24464 | 7.293848 | 1.320438 | 1 | 0.250513 | 1 |
| Bacteroidetes | 164179 | 50 | -0.04668 | 8.088195 | 0.072674 | 1 | 0.787482 | 1 |
| Candidatus_Saccharibacteria | 7228 | 49 | -0.58341 | 4.823741 | 2.749729 | 1 | 0.097271 | 0.778168 |
| Spirochaetes | 8455 | 41 | 0.873865 | 5.431663 | 2.257937 | 1 | 0.132931 | 0.930517 |
| SR1 | 958 | 25 | 0.070555 | 3.564453 | 0.012924 | 1 | 0.909489 | 1 |
| Tenericutes | 839 | 13 | 1.90964 | 4.562009 | 6.751342 | 1 | 0.009368 | 0.093677 |
| Synergistetes | 452 | 10 | 0.12705 | 3.963453 | 0.046024 | 1 | 0.830132 | 1 |
| Bacteria..unc.. | 257 | 17 | -0.01461 | 2.611566 | 0.00053 | 1 | 0.98163 | 1 |
|  |  |  |  |  |  |  |  |  |
| **Healthy Controls versus Ocrelizumab-treated Multiple Sclerosis patients** | | | | | |  |  |  |
| Phylum | Abundance | Prevalence | log2FoldChange | baseMean | Chisq | df | P-value | P adjusted |
| Firmicutes | 668315 | 57 | -0.04549 | 9.36097 | 0.444712 | 1 | 0.504857 | 1 |
| Actinobacteria | 163182 | 57 | -0.39717 | 7.847513 | 4.805058 | 1 | 0.028376 | 0.255387 |
| Proteobacteria | 196354 | 57 | 0.523197 | 8.18195 | 6.270601 | 1 | 0.012276 | 0.122757 |
| Fusobacteria | 73580 | 57 | -0.32497 | 7.137262 | 2.665884 | 1 | 0.102521 | 0.820167 |
| Bacteroidetes | 162862 | 57 | -0.04007 | 7.975059 | 0.057684 | 1 | 0.810194 | 1 |
| Candidatus_Saccharibacteria | 8883 | 55 | 0.305325 | 5.151675 | 1.112659 | 1 | 0.291505 | 1 |
| Spirochaetes | 7956 | 45 | 0.744562 | 5.309357 | 2.509481 | 1 | 0.113163 | 0.820167 |
| SR1 | 1042 | 25 | 0.80037 | 3.816885 | 1.515573 | 1 | 0.218291 | 1 |
| Tenericutes | 769 | 19 | 0.80515 | 3.770086 | 1.651238 | 1 | 0.198791 | 1 |
| Synergistetes | 528 | 14 | 0.037493 | 3.755169 | 0.003931 | 1 | 0.950005 | 1 |
| Bacteria..unc.. | 419 | 16 | 1.736777 | 3.429242 | 7.070636 | 1 | 0.007836 | 0.086193 |

**Suppl. Table 4**

| **Diversity in stool samples during treatment** | | |  |  |  |  |  |
| --- | --- | --- | --- | --- | --- | --- | --- |
|  |  |  |  |  |  |  |  |
| **Alpha-diversity** |  |  |  |  |  |  |  |
| Group | DataName | Method | After | Before | Statistic | EffectSize | P-value |
| After(TP2) vs Before(TP1) | Chao1 by Group | Paired t-test | 11 | 11 | 0.285526 | 0.147509 | 0.78106 |
| After(TP2) vs Before(TP1) | Shannon by Group | Wilcoxon signed rank test | 11 | 11 | 34 | 0.026808 | 0.96582 |
| After(TP2) vs Before(TP1) | InvSimpson by Group | Wilcoxon signed rank test | 11 | 11 | 32 | 0.026808 | 0.96582 |
| After(TP3) vs Before(TP1) | Chao1 by Group | Paired t-test | 10 | 10 | 1.063938 | 0.232598 | 0.31506 |
| After(TP3) vs Before(TP1) | Shannon by Group | Wilcoxon signed rank test | 10 | 10 | 17 | 0.338446 | 0.32226 |
| After(TP3) vs Before(TP1) | InvSimpson by Group | Wilcoxon signed rank test | 10 | 10 | 16 | 0.370679 | 0.27539 |
| After(TP4) vs Before(TP1) | Chao1 by Group | Paired t-test | 6 | 6 | 1.765618 | 0.952763 | 0.13772 |
| After(TP4) vs Before(TP1) | Shannon by Group | Wilcoxon signed rank test | 6 | 6 | 8 | 0.21398 | 0.6875 |
| After(TP4) vs Before(TP1) | InvSimpson by Group | Wilcoxon signed rank test | 6 | 6 | 7 | 0.299572 | 0.5625 |
|  |  |  |  |  |  |  |  |
| **Beta-diversity** |  |  |  |  |  |  |  |
|  |  |  |  |  |  |  |  |
| Groups | Test | Distance | Df | SumOfSqs | R2 | F | P-value |
| After (TP2) vs Before(TP1) | Adonis2 | Bray | 1 | 0.234936 | 0.020365 | 0.60285 | 0.982 |
| After (TP3) vs Before(TP1) | Adonis2 | Bray | 1 | 0.242845 | 0.021337 | 0.632263 | 0.991 |
| After (TP4) vs Before(TP1) | Adonis2 | Bray | 1 | 0.350794 | 0.039684 | 0.909118 | 0.682 |
|  |  |  |  |  |  |  |  |
| Status | Test | Distance | Permutations | Statistic.R |  |  | P-value |
| MS-O_TP1 vs Healthy | Anosim | Bray | 999 | 0.128774 |  |  | 0.036 |
| MS-O_TP2 vs Healthy | Anosim | Bray | 999 | 0.181914 |  |  | 0.005 |
| MS-O_TP3 vs Healthy | Anosim | Bray | 999 | 0.12957 |  |  | 0.047 |
| MS-O_TP4 vs Healthy | Anosim | Bray | 999 | 0.203488 |  |  | 0.028 |
|  |  |  |  |  |  |  |  |
|  |  |  |  |  |  |  |  |
| **Diversity in swab samples during treatment** | | |  |  |  |  |  |
|  |  |  |  |  |  |  |  |
| **Alpha-diversity** |  |  |  |  |  |  |  |
| Group | DataName | Method | After | Before | Statistic | EffectSize | P-value |
| After(TP2) vs Before(TP1) | Chao1 by Group | Paired t-test | 15 | 15 | 0.684579 | 0.273299 | 0.504786 |
| After(TP2) vs Before(TP1) | Shannon by Group | Wilcoxon signed rank test | 15 | 15 | 51 | 0.131982 | 0.638672 |
| After(TP2) vs Before(TP1) | InvSimpson by Group | Wilcoxon signed rank test | 15 | 15 | 50 | 0.146647 | 0.599487 |
| After(TP3) vs Before(TP1) | Chao1 by Group | Paired t-test | 15 | 15 | 1.589986 | 0.463225 | 0.134159 |
| After(TP3) vs Before(TP1) | Shannon by Group | Wilcoxon signed rank test | 15 | 15 | 69 | 0.131982 | 0.638672 |
| After(TP3) vs Before(TP1) | InvSimpson by Group | Wilcoxon signed rank test | 15 | 15 | 65 | 0.073324 | 0.803955 |
| After(TP4) vs Before(TP1) | Chao1 by Group | Paired t-test | 12 | 12 | 2.595964 | 0.95015 | 0.024872 |
| After(TP4) vs Before(TP1) | Shannon by Group | Wilcoxon signed rank test | 12 | 12 | 49 | 0.226455 | 0.469727 |
| After(TP4) vs Before(TP1) | InvSimpson by Group | Wilcoxon signed rank test | 12 | 12 | 38 | 0.022646 | 0.969727 |

Supplementary Figure S1

**
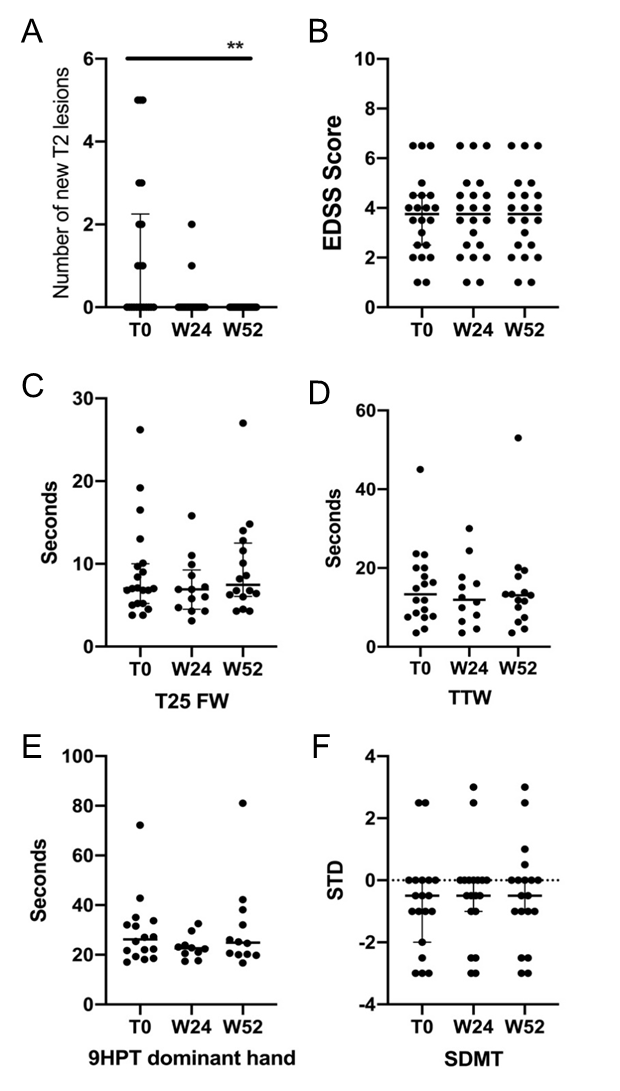
**

**Figure S1:**  *MRI and clinical outcomes during the 12-month follow-up of the MS-O patient group.* While the number of new T2 lesions declines swiftly (A), EDSS and MSFC outcomes are stable throughout the follow-up.in all samples (B-F).
